# Cryo‐EM reveals an ensemble of cytochrome P450 reductase conformations in solution

**DOI:** 10.1002/pro.70448

**Published:** 2026-01-21

**Authors:** Galina I. Lepesheva, Tatiana Y. Hargrove, Yi Ren

**Affiliations:** ^1^ Department of Biochemistry Vanderbilt University School of Medicine Nashville Tennessee USA; ^2^ Center for Structural Biology Vanderbilt University Nashville Tennessee USA

**Keywords:** cryogenic electron microscopy, cytochrome P450, cytochrome P450 reductase, electron transfer, protein structure

## Abstract

The eukaryotic electron transport system, mediated by cytochrome P450 reductase (CPR), plays a crucial role in driving myriads of reactions involved in the biosynthesis of physiologically active compounds (such as sterols, steroids, vitamins, and natural products), as well as in the metabolism of drugs, toxins, and carcinogens. CPR is a diflavin‐containing enzyme found ubiquitously on the cytosolic side of the endoplasmic reticulum. While several crystal structures of CPR are available, its conformational states in solution, along with the molecular details of action, remain debatable. Here, we determined the 3.3 Å cryo‐EM structure of rat CPR, marking the first electron microscopy structure of this relatively small protein (77 kDa). In this structure, the full‐length, fully active enzyme adopts a compact conformation, which, however, is more relaxed than in crystal structures. Moreover, we structurally characterized less populated variations of compact CPR conformations and identified a fraction of molecules (~20%) with the FMN‐binding domain either not visible or positioned far from the rest of the catalytic core. These results support the idea that large‐scale interdomain rearrangements serve as the structural basis for CPR function and suggest that cryo‐EM techniques can help uncover the intricate molecular mechanisms governing the CPR‐mediated electron transfer cycle.

## 1 INTRODUCTION

**FIGURE 1:**
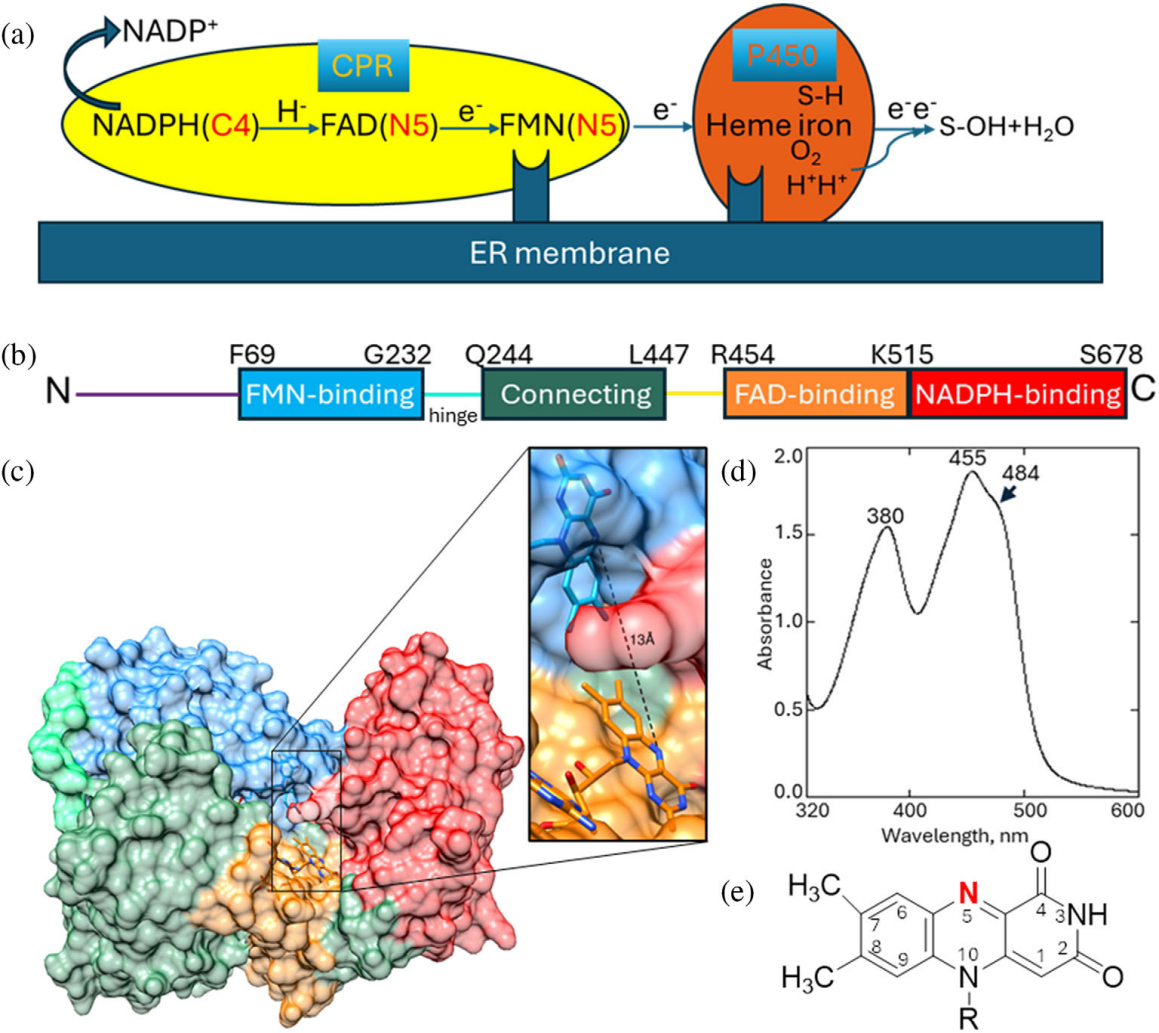
Structural organization of CPR. (a) Schematic representation of a CPR‐mediated ET chain with cytochrome P450 as an example of an electron acceptor. (b) Linear domain arrangement, from the N‐ to the C‐terminus, rat CPR numbering. (c) 3D domain arrangement in the same color code (1JA0‐based); carbon atoms of FMN and FAD are blue and orange, respectively. (d) Absolute absorption spectrum of the purified CPR sample, with the characteristic for the oxidized state maxima at 380 and 455 nm and a shoulder at 484 nm. Upon reduction of the FAD and FMN cofactors to the semiquinone and hydroquinone species, the absorption of both peaks decreases, with a new maximum appearing at 585 nm (for semiquinones). (e) Isoalloxazine ring of FAD/FMN in the oxidized quinone state. The atoms are numbered, the N5 atom highlighted in red.

Electron transfer (ET) is a fundamental process in biology. It is essential for energy conversion and various metabolic pathways, powering long‐chain electron transport systems, such as photosynthesis and mitochondrial respiration, as well as diverse small‐scale redox reactions. Cytochrome P450 reductase (CPR, EC 1.6.2.4) is the key eukaryotic ET protein that receives electrons from NADPH and transfers them to all microsomal cytochromes P450, enabling them to perform their functions (Figure 1a). Other physiological CPR electron acceptors are squalene monooxygenase, heme oxygenase, cytochrome b5, fatty acid desaturase, and some anticancer drugs (Pandey and Flück 2013). There is only one CPR gene in non‐photosynthetic organisms, whereas some plants have up to three genes (Jensen and Møller 2010), and the same CPR molecule supports catalysis of multiple acceptor molecules, for example, in the liver microsomes, P450s outnumber CPR by 5‐20 to 1 (Reed et al. 2006; Reed and Backes 2012).

Structurally, CPR is a diflavin‐containing enzyme with a molecular weight between 75 and 81 kDa (Masters and Okita 1980; Murataliev et al. 2004). It is anchored to the cytoplasmic surface of the smooth endoplasmic reticulum (ER) via the N‐terminal sequence of ~6 kDa that is followed by the catalytic core (Figure 1b,c). The core consists of four segments (Wang et al. 1997), named the FMN‐binding domain, the connecting domain, the FAD‐binding domain, and the NADPH‐binding domain, from the N‐ to the C‐terminus. Based on the sequence homology analysis, it has been suggested that CPR may have evolved as a fusion of two ancestral bacterial genes, encoding two flavin‐containing ET enzymes (Porter and Kasper 1985, 1986), possibly to achieve higher ET efficiency (Murataliev et al. 2004). Indeed, the Protein Data Bank (PDB) search shows that the FMN‐binding domain is structurally similar to bacterial FMN‐containing flavodoxins, and the FAD/NADPH‐binding portion resembles bacterial NAD(P)H‐dependent FAD‐containing oxidoreductases (Figure S1, Supporting Information). The connecting domain that links the FMN and FAD domains is unique to CPR. It begins with the long loop, often referred to as a hinge, which comprises 13 amino acid residues (G232‐Q244) in rat CPR.

The ET chain (Figure 1a) starts with a two‐electron hydride anion transfer from the C4 atom of the nicotinamide part of NADPH to the redox center of FAD, specifically the N5 atom of the isoalloxazine ring (Figure 1e). Then it proceeds as a single electron transfer to the N5 atom of the isoalloxazine ring of FMN and next from FMN to the redox center of a partner protein, such as ferric and oxyferrous iron of the heme in cytochromes P450, which use the electrons to split molecular oxygen, oxidizing their substrates and forming a molecule of water (Hargrove et al. 2020). The CPR‐mediated ET process is of great importance because it regulates the synthesis of various biologically active compounds, including sterols, steroid hormones, and vitamins. It also enables the oxidative metabolism of drugs, environmental pollutants, and carcinogens, as well as processes such as fatty acid desaturation and elongation, heme degradation, and others.

To date, several crystal structures of CPR from six organisms have been reported, including four non‐photosynthetic species and two plants. All the protein constructs, however, lack the membrane‐anchoring N‐terminus, which is known to be necessary for CPR activity (Backes and Kelley 2003; Xia et al. 2019), but had to be truncated for crystallization because of its highly hydrophobic nature. The first crystal structure determined was that of the truncated wild‐type rat CPR (Δ63, the hinge loop missing 7 residues (236–242); PDB code 1AMO, 2.6 Å) (Wang et al. 1997). This was followed by the structure of Δ47 CPR from *Saccharomyces cerevisiae* (PDB code 2BN4, 2.9 Å; Lamb et al. 2006), Δ69 CPR from human (PDB code 3QE2, 1.75 Å; Xia et al. 2011), and Δ45 CPR from *Candida tropicalis* (PDB code 6T1U, 1.5 Å; Ebrecht et al. 2019).

Despite the low amino acid sequence identity of the proteins (~40%), the structures display remarkable similarity, adopting essentially the same “closed” conformation, which is consistent with intramolecular (FAD‐to‐FMN), but not intermolecular (FMN‐to‐P450) ET. The root mean square deviation (RMSD) range between their Cα atoms is only 0.7–1.1 Å. Moreover, all single amino acid mutants that have been crystallized, including clinically relevant mutants of human CPR, were also found in this closed conformation, although with some subtle differences affecting cofactor binding, thermostability, and other properties (Hubbard et al. 2001; McCammon et al. 2016; Rwere et al. 2016; Xia et al. 2011). The distance between the N5 atoms in the FAD and FMN isoalloxazine rings (N5‐to‐N5) in all these structures remains strictly 13 Å.

In this closed conformation, CPR cannot perform intermolecular ET because its FMN cofactor is buried inside the protein (Figure 1c), being positioned next to the NADPH‐binding domain and embedded in its crevice so that the distance between FMN and the P450 heme iron cannot become sufficiently close—at least 17 Å (Marcus and Sutin 1985)—unless some changes occur. Indeed, two CPR structures in completely different conformations were determined. One of them was a chimera composed of the yeast FMN‐binding domain (residues 44–211) and the other three domains of human CPR (residues 232–677), PDB code 3FJO, 2.5 Å (Aigrain et al. 2009). In this structure, the FMN domain is located far away from the rest of the molecule, with the FMN isoalloxazine ring exposed to the solvent, and the N5‐to‐N5 distance increased to 83 Å. The authors suggested that the chimeric protein may have adopted the open conformation because of the differences (e.g., redox potentials) between human and yeast CPR (Aigrain et al. 2009). The other structure was of the Δ56 rat CPR mutant, which has four of the 13 residues in the hinge loop deleted (ΔT236GEE239), PDB code 3ES9, 3.4 Å (Hamdane et al. 2009). In molecule A (the one with a complete electron density map in this structure), the FMN binding domain is rotated relative to the rest of the catalytic core. As a result of this rotation, the isoalloxazine ring of FMN is also exposed to the solvent, and the N5‐to‐N5 distance becomes 32 Å. This mutant was subsequently co‐crystallized in complex with heme‐bound rat heme oxygenase I, and the CPR molecule displayed a conformation very similar to that of molecule A in 3ES9, with the N5‐to‐N5 distance of 30 Å (Sugishima et al. 2014). The distance between the acceptor heme iron and the N5 atom of FMN was 14 Å, allowing direct intermolecular ET.

The concept that the ET cycle involves large‐scale conformational transitions between closed and open states of CPR offered a plausible explanation for the enzyme functioning. Various techniques have been employed to explore this idea, including nuclear magnetic resonance (Ellis et al. 2009; Huang et al. 2013; Vincent et al. 2012), small‐angle x‐ray scattering (Ellis et al. 2009; Frances et al. 2015), small‐angle neutron scattering (Freeman et al. 2017; Huang et al. 2013), ion mobility mass spectrometry (Jenner et al. 2011), neutron reflectometry (Wadsäter et al. 2012), fluorescence resonance energy transfer (Bavishi et al. 2018; Kovrigina et al. 2016; Zhang et al. 2022a), double electron–electron resonance (Bizet et al. 2024), as well as theoretical computational approaches (Mukherjee et al. 2021; Xia and Hirao 2024). These studies have yielded different results, leading to various, sometimes controversial hypotheses. Experimental molecular information about functional CPR in solution has been missing.

The goals of our work were to determine the rat CPR structure by cryogenic electron microscopy (cryo‐EM) and to investigate whether the technique can be used to distinguish between different conformational states of the enzyme.

## 2 RESULTS

A full‐length rat CPR construct, comprising all 678 amino acid residues (77 kDa), was used in the experiments. The protein was purified in its oxidized form (Figure 1d) and demonstrated consistent catalytic activity (supporting the turnover of human P450 sterol 14α‐demethylase (CYP51) of approximately 50 nmol/nmol/min) (Hargrove et al. 2016) along with good biological stability. After vitrification, the sample was subjected to EM imaging. The movies were corrected for motion, and a defocus landscape was assessed through patch‐based contrast transfer function (CTF) estimation. CPR particles were readily discernible in EM images due to their characteristic shape. Two‐dimensional (2D) classification of automatically picked particles generated class averages with recognizable secondary structure features. Details on the data processing workflow are presented in Figure S2.

### 2.1 The 3.3 Å cryo‐EM map and structure (9EF0)

**FIGURE 2:**
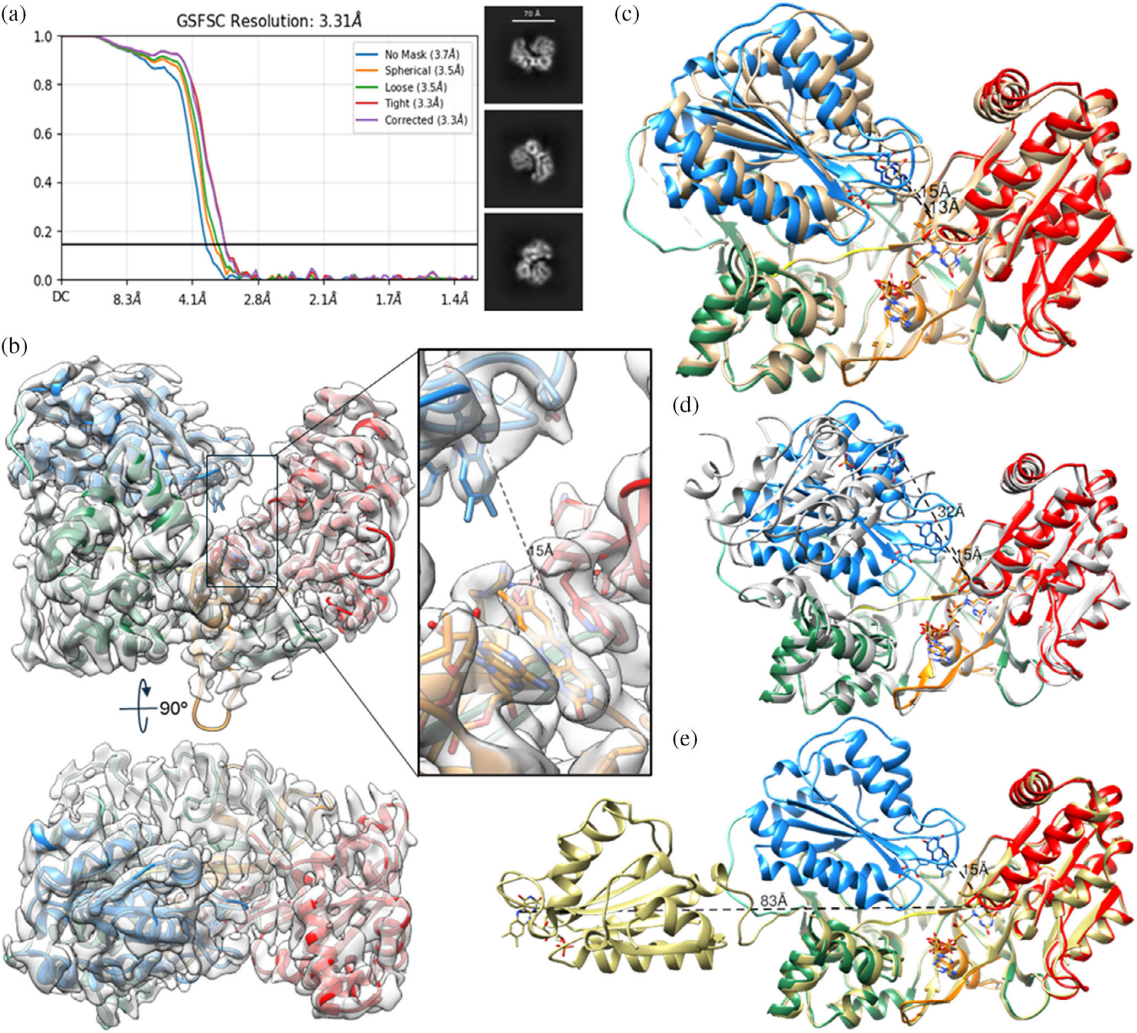
Cryo‐EM density map EMD‐40857 and 9EF0 structure of rat CPR. (a) The gold‐standard Fourier shell correlation (FSC) plots of the final map reconstruction and representative 2D class averages. (b) Overlaid EM density and atomic model, the map is semitransparent gray, the color code for the structure is the same as in Figure 1b,c. The inset shows the electron density and some protein side chains around FMN and FAD. (c–e) The EM structure 9EF0 superimposed with the crystal structures 1AMO (tan), 3ES9 (molecule A, gray), and 3FJO (khaki), respectively. The corresponding N5 (FAD) to N5 (FMN) distances are marked.

Although cryo‐EM techniques are rapidly advancing, enabling the generation of near‐atomic resolution structures and overcoming size‐limitation barriers, applying cryo‐EM to determine the three‐dimensional structures of small proteins (<100 kDa) remains challenging. Therefore, we initially focused on selecting only the best classes of particles to achieve the highest possible resolution. After several rounds of 2D classifications, ab‐initio model reconstruction, heterogeneous and homogeneous refinements (as detailed in section 4), we obtained an electron density map with an average resolution of 3.3 Å (Figure 2a and Table 1). The mutant rat structure 3ES9, molecule A (Hamdane et al. 2009) was then used to build the structural model. We chose it because we believed this conformation was plausible in the solution where CPR performs both intramolecular and intermolecular ET, and because this was the conformation found in the structure of CPR complexed with heme oxygenase (Sugishima et al. 2014). However, when the 3ES9 molecule was docked into the EM density map, we observed that while the fit for the C‐terminal segments of CPR was good (starting from the connecting domain, Y245, the beginning of the β6 strand (245–250); Wang et al. 1997), the FMN‐binding domain was clearly misplaced (Figure S3) and needed to be rotated approximately 90° around the β6 strand and swung forward along the hinge loop, which in wild‐type CPR is four residues longer than it is in the 3ES9 mutant. The FMN‐binding domain was then deleted from the 3ES9 structure and fitted into the EM density map separately. When the model was refined (Figure 2b), we found that, except for the repositioning of the FMN‐binding domain and altered conformation of the hinge loop, there were no substantial changes in the arrangement of the secondary structural elements, especially within the domains.

**TABLE 1:** Cryo‐EM data collection, refinement, and validation statistics.

Data collection and processing	
Microscope/detector	Titan Krios G4/Gatan K3
Magnification	130,000×
Voltage (kV)	300
Electron exposure (e Å^−2^)	53
Defocus range (μm)	−0.8 to −2.4
Pixel size (Å)	0.647
Symmetry imposed	C1
No. of initial particle images	1,595,326
No. of final particle images	671,545
Map resolution (Å)	3.31 (3.76)
FSC threshold	0.143 (0.5)

Refinement	
Initial model used	de novo
Model resolution (Å)	3.33
Map sharpening B factor (Å^2^)	181.2
Model composition	
Non‐hydrogen atoms	4909
Protein residues	613
Ligands	2
Average B factor (Å^2^)	92.8
r.m.s. deviations	
Bonds (Å)	0.002
Angles (°)	0.538

Validation	
MolProbity score	1.86
Clashscore	10.5
Poor rotamers (%)	1.3
Ramachandran plot	
Favored (%)	96.56
Allowed (%)	3.44
Disallowed (%)	0

Overall, the EM structure was more similar to the “closed” crystal structures of the wild‐type CPRs than to the open structure of the ΔTGEE‐hinge truncated mutant (3ES9). However, the “closed” EM structure appeared more relaxed, with the distance between the N5 atoms of FAD and FMN increased from 13 to 15 Å (Figures 2c and S4). Structural alignment analysis revealed an average root mean square deviation (RMSD) of 1.2 Å between the Cα atoms in the EM structure (9EF0) and crystal structure 1AMO. In comparison, the RMS XYZ displacement values for the Cα atoms of residues 66–670 in 3ES9 and 3FJO (yeast/human chimera) versus 9EF0 were 9.2 and 29.8 Å, respectively, due to the differences in the position of the FMN‐binding domain (Figure 2d,e). For the rest of the molecule (residues 238–678), the RMSDs were 0.75 Å (1AMO) and 0.74 Å (3ES9 and 3FJO). Interestingly, the RMSD with the AlphaFold model of wild‐type rat CPR (AF_P00388_F1) was 1.05 Å (not shown).

Even though we used the full‐length protein in the experiments, electron density was only observed from residue 66 onward, that is, from the ‐E66S67S68‐loop segment that precedes the A helix in the FMN‐binding domain (F69‐V233). The first 65 amino acids, which include the N‐terminal membrane anchoring helix and most of the linker loop connecting the catalytic core of the protein to the membrane (Xia et al. 2019), were not detectable. This absence of density suggests that the N‐terminus of CPR is highly flexible in solution, further supporting findings from site‐directed mutagenesis studies (Rwere et al. 2024). As expected, there was no traceable density for the NAPD(H) molecule in the structure. We did not add NADPH during purification to ensure that all CPR molecules contained FAD and FMN cofactors in the same oxidation state, specifically the quinone form (shown in Figure 1e).

**FIGURE 3:**
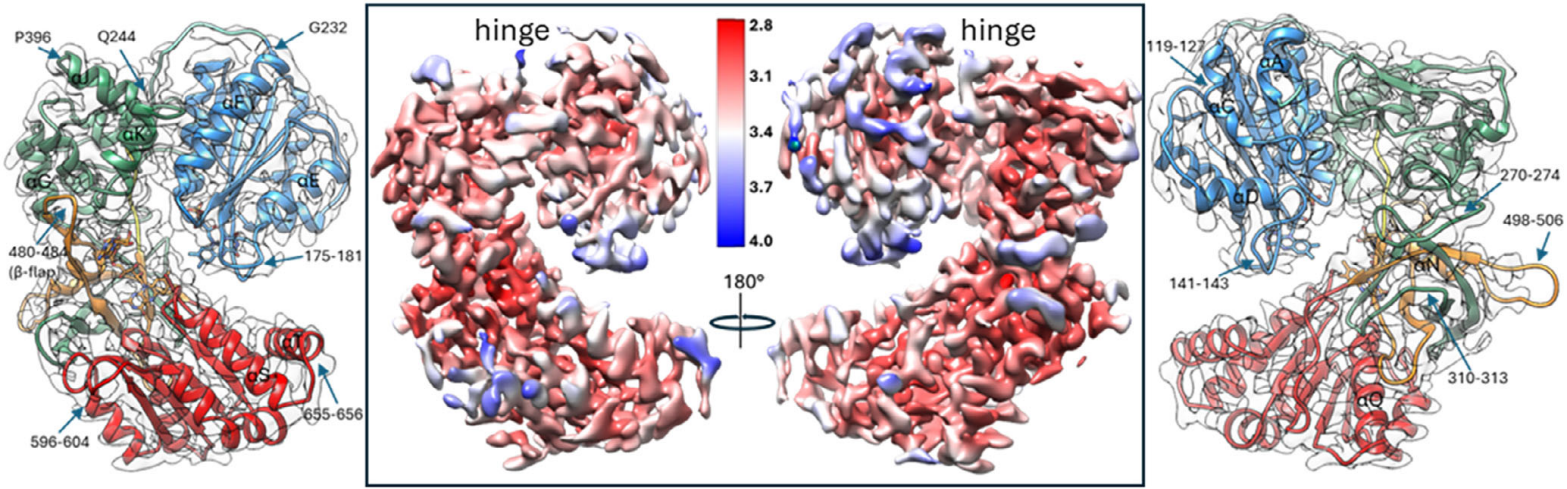
Local resolution of the CPR cryo‐EM map (EMD‐40587) scaled between 4.0 and 2.8 Å: two side views, re‐face (left), and si‐face (right). The protein ribbon within the semitransparent map oriented the same way is shown alongside, with the most flexible segments as well as some helices marked for clarity.

The local resolution density map, constructed in the range of 2.8–4.0 Å, reflects the relative rigidity of the CPR polypeptide chain (Figure 3). The most rigid regions are seen inside the protein globule, and the most flexible areas are the loops, especially the hinge loop (residues 232–244) and the loop between helix N and β strand 16 (Wang et al. 1997) (residues 498–506), where some parts drop below the specified resolution range. While the high plasticity of the hinge was expected, the reason for the elevated mobility of the 498–506 loop, also known as the tip of the β‐finger (Xia et al. 2011), located in the FAD‐binding area, remains unknown. Overall, the highest flexibility is exhibited by the FMN‐binding domain, particularly on the si‐face of the CPR molecule. This aligns with the idea that the functionally essential conformational dynamics of CPR involve interdomain motions and that the hinge loop at residues 232–244 is likely to govern these movements, as was indirectly shown by Campelo et al. (2018).

### 2.2 Variability of closed CPR conformations

**FIGURE 4:**
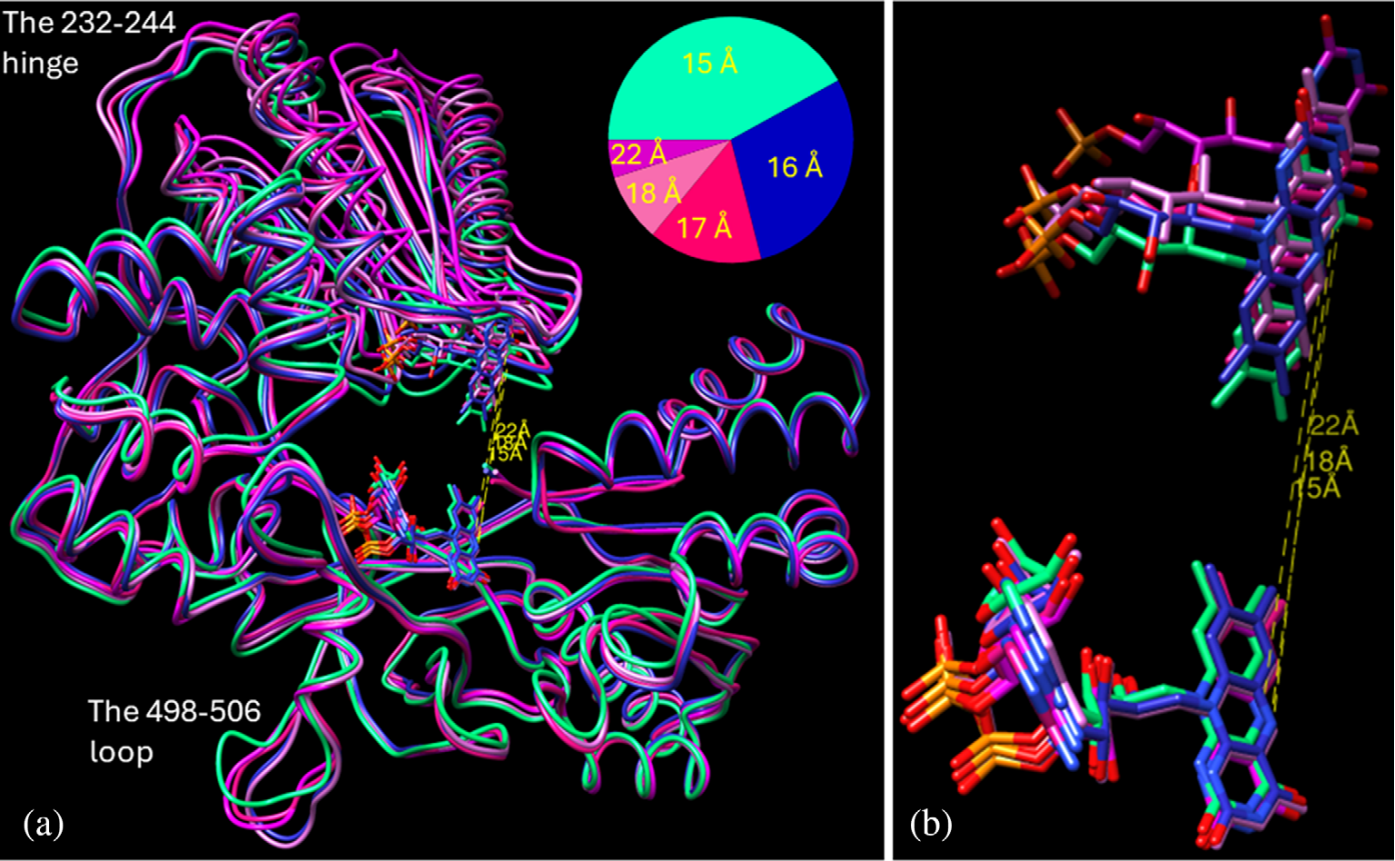
An ensemble of closed CPR conformations. (a) Five superimposed molecules in licorice representation. The slice diagram in the same color code shows fractions of particles used in the final EM map reconstructions. The corresponding FAD‐FMN N5‐to‐N5 distances are displayed in yellow. (b) Enlarged view of the cofactors. Separate images of each structure within its EM map are shown in Figure S5.

The processed dataset (2.9 million selected particles, 4.9 Å electron density map after homogeneous refinement) (Figure S2) was then used to search for conformational heterogeneity. At least five distinct variations of the “relaxed” closed CPR conformations were identified, totaling 1,595,326 particles after final refinement. These variations exhibited the FAD–FMN N5‐to‐N5 distances of 15 Å (9EF0, ~42% of particles), 16 Å (~29%), 17 Å (~15%), 18 Å (~9%), and 22 Å (~5%) (Figure 4). The greatest variability was observed in the FMN‐binding domain, including the hinge, and in the 498–506 loop (the tip of the β‐finger). The average resolutions of the electron density maps at the FSC cut‐off of 0.143 were 3.3 Å (9EF0), 3.5 Å, 4.3 Å, 3.6 Å, and 4.4 Å (Figure S5). The RMSDs of Cα atoms between 9EF0 and the other EM structures were 0.6 Å, 1.2 Å, 1.4 Å, and 1.5 Å, respectively (see also Movie S1).

The data indicate that, in solution, the conformational landscape of oxidized CPR is primarily shaped by molecular forms that differ from one another while maintaining relative positions of the domains that are consistent with intramolecular ET. When crystallized, the FMN‐binding domain and the rest of the molecule are pulled even closer together to achieve uniformity, causing the N5‐to‐N5 distance to decrease to 13 Å.

### 2.3 CPR molecules in alternative conformations

**FIGURE 5:**
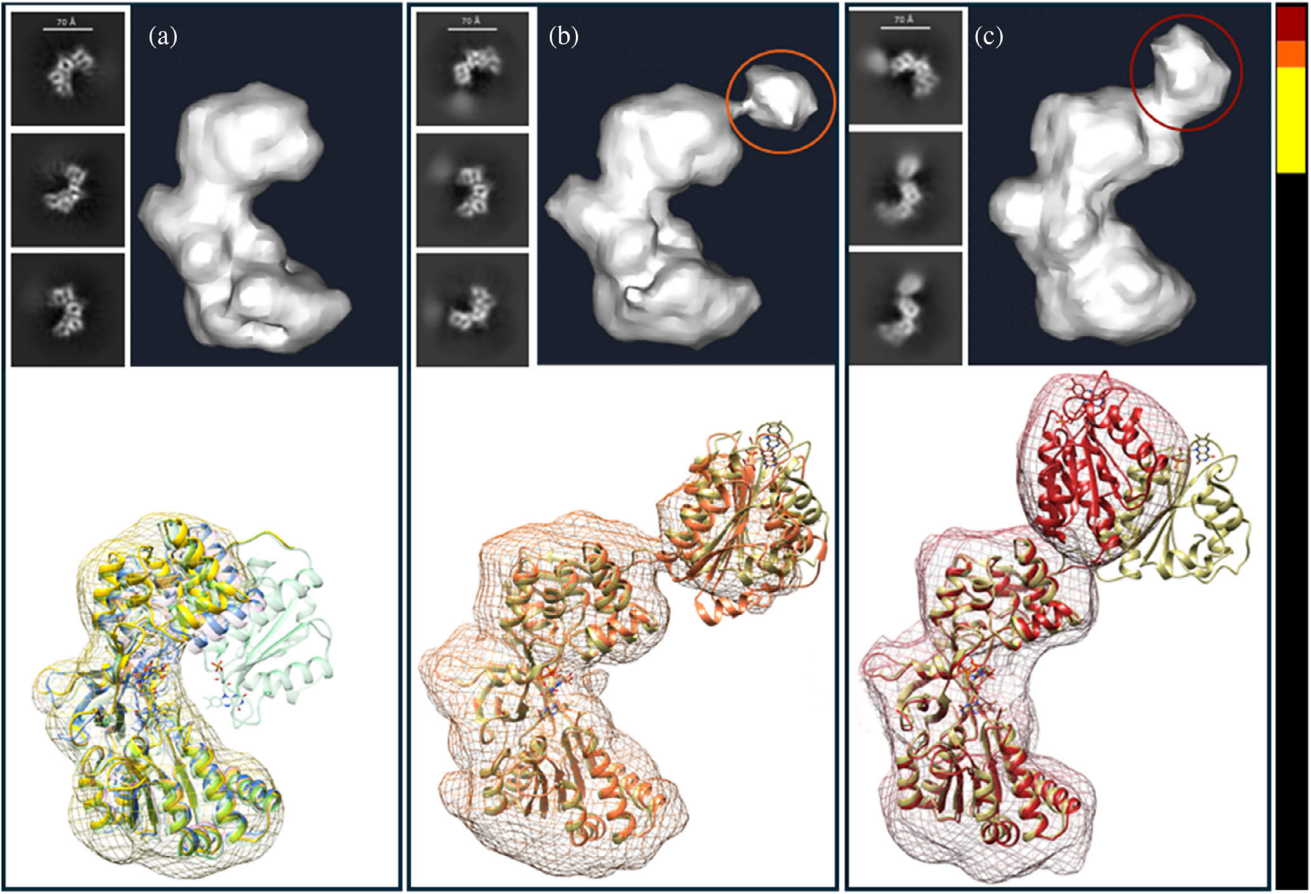
Alternative conformational states of oxidized CPR. (a) Particles without density for the FMN‐binding domain. (b, c) Particles with some density for the FMN‐binding domain (circled) in open conformations. Top: representative 2D class averages and the corresponding ab‐initio reconstruction volumes from Cryosparc. Bottom: ribbon representations of the refined structural models in their EM density maps (mesh), yellow, orange, and firebrick, respectively. (a) Superimposed with the EM structure 9EF0 (green) and the crystal structures of CPR from *Arabidopsis thaliana* (5GXU, molecule B, pink) and *Sorghum bicolor* (7SUZ, blue). (b, c) Superimposed with the crystal structure of human/yeast chimeric CPR 3FJO (khaki). The slice diagram on the right shows the fractions of particles in the preferred closed conformations (black, 81% in total) versus open conformations (19% in total: a, 12%; b, 3%; c, 4%).

During data processing, a few classes of smaller, oddly shaped particles were noticed. They appeared to lack the FMN‐binding domain completely and were initially excluded. These classes were then used as templates for a new round of particle picking, followed by Topaz training, 2D classification, ab initio reconstruction (Figure 5a, top), refinements, and model fitting. The 2D class averages and the FSC plots for the final map reconstruction, which included 225,003 particles and achieved an average resolution of 4.5 Å, are shown in Figure S6a. As anticipated, there was no density for the FMN‐binding domain, while the fit of the rest of the 9EF0 structure was good (Figure 5a, bottom). Overall, the molecule resembled the crystal structures of plant CPRs, from *Arabidopsis thaliana* (5GXU, pink ribbon in Figure 5a; Niu et al. 2017) and *Sorghum bicolor* (7SUZ, blue ribbon in Figure 5a; Zhang et al. 2022b), both of which also lack the FMN‐binding domain, with the RMSD between Cα atoms in the rest of the molecule being 1.9 and 2.0 Å, respectively. In comparison, the RMSD between Cα atoms of 5GXU and 7SUZ is 1.3 Å. Since the protein sample used for the cryo‐EM experiments was electrophoretically pure and showed no signs of proteolytic degradation, we conclude that the maps lacking FMN‐domain density represent alternative conformational states of CPR with varying domain positions, as proposed for the aforementioned crystal structures of plant CPRs (Zhang et al. 2022b). In fact, two such alternative (open) conformations have been captured through further data processing, although only low‐resolution maps (~6.4 Å) were reconstructed (Figure S6b,c). One set, consisting of 44,400 particles, produced a map that showed a relatively good fit for the position of the FMN‐binding domain found in crystal structure 3FJ0 (human/yeast chimeric CPR) (Figure 5b). The second dataset, which included 69,108 particles, suggested a similar spatial location of the FMN‐binding domain but with a rotation of approximately 180° compared to its orientation in 3FJ0 (Figure 5c).

## 3 DISCUSSION

Using cryo‐EM, we have determined for the first time a high‐resolution structure of fully functional wild‐type CPR and outlined a structural ensemble that reflects the conformational landscape of this enzyme in solution, thus providing data which are more relevant to physiological conditions. The results support the idea that CPR operates by alternating between closed and open conformations. They also imply that the open states must resemble the 3FJO (chimeric) rather than the 3ES9 structure. Furthermore, the three‐domain C‐terminal region of the catalytic core must be the part that moves, as in vivo the FMN‐binding domain is anchored to the ER membrane. The length and flexibility of the hinge loop between the domains probably play a crucial role, and we assume that the rat CPR mutant with the truncated hinge region (3ES9) lost its ability to transfer electrons from FAD to FMN, as demonstrated by Hamdane et al. (2009), because the mutant protein is “trapped” in this open conformation, preventing the two flavin cofactors from approaching each other. Finally, the presence of molecules with potentially opposing orientations of the FMN‐binding domain suggests that, in addition to the opening and closing motions, rotations of the FMN domain—previously proposed through NMR studies (Huang et al. 2013) and molecular dynamics simulations (Sündermann and Oostenbrink 2013) for the 3ES9‐like models of the enzyme—must also be involved in the functionally essential conformational dynamics of CPR. The relative abundance of open and closed conformational populations, which was approximately 1–4 under our experimental conditions, is likely to vary depending on factors such as electron availability, the proximity and redox potential of an acceptor protein, and possibly other specific environmental features (e.g., membrane environment as demonstrated by Campelo et al. 2018).

**FIGURE 6:**
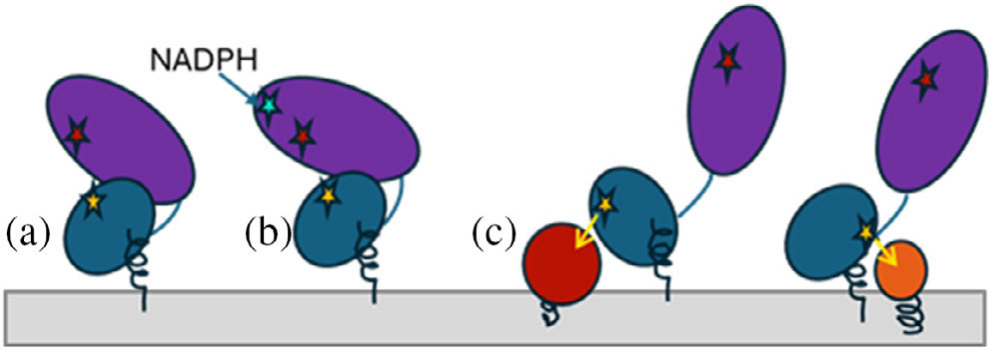
A cartoon representation of the proposed conformational rearrangements in the CPR molecule during the ET process. (a, b) Closed conformations (intramolecular transfer); (c) open conformations (intermolecular transfer). The FMN‐binding domain is shown in blue, and the rest of the catalytic core is purple. NADPH, FAD, and FMN are represented as cyan, red, and yellow stars, respectively. Electron acceptors are depicted as red and orange circles.

In summary, single‐particle analysis shows that in its oxidized state in physiological solution, full‐length wild‐type CPR primarily exists as an ensemble of closed conformations (see Figure 4). These conformations are consistent with intramolecular electron transfer from FAD to FMN, though they are more relaxed compared to the closed conformation seen in crystal structures (with an N5‐to‐N5 distance range of 15–22 Å versus 13 Å, respectively). We surmise that the binding of NADPH may cause CPR to adopt a tighter conformation, thereby promoting more efficient intramolecular ET (Figure 6a,b). When the reduced FMN “senses” an electron acceptor partner (e.g., substrate‐bound cytochrome P450), the membrane‐tethered FMN‐binding domain forces the cytosolic rest of the CPR molecule away and transfers the electron to the acceptor. The domain movement is likely to include both swinging back and forth of the FAD/NADPH‐binding portion and pivoting of the FMN‐binding domain around the membrane anchor towards the neighboring redox partner of the highest affinity (Figure 6c). The latter should facilitate sampling of electron transfer from the same CPR molecule to various neighboring membrane‐bound redox partner proteins. When electrons are transferred, CPR returns to its relaxed, oxidized state until it binds another NADPH molecule. Our data suggest that cryo‐EM techniques can be successfully applied to track conformational transitions that occur in CPR during the ET process, which, in turn, should facilitate understanding the interplay between the electron donor and other electron transfer chain components.

## 4 METHODS

### 4.1 CPR expression and characterization

Recombinant full‐length rat CPR was expressed in the DH5α strain of *Escherichia coli* and purified as described (Hanna et al. 1998). Purity and the absence of proteolytic degradation were confirmed by polyacrylamide gel electrophoresis (PAGE). Absolute absorbance spectra were recorded using a dual‐beam Shimadzu UV‐2600i spectrophotometer throughout the entire procedure to ensure that the protein remained in its oxidized (di‐quinone FAD, FMN) state. Enzymatic activity was confirmed in the assay with human sterol 14α‐demethylase (CYP51) as described previously (Hargrove et al. 2016).

### 4.2 Sample preparation

To prepare the cryo‐EM samples, CPR was diluted right before use to a working concentration with PBS (Gibco) containing 5% glycerol. The protein was then deposited on glow‐discharged, holey carbon cryo‐EM grids (Quantifoil, R1.2/1.3, 300 mesh Cu). The grids were blotted for 6 s with a blotting force of 12 at 4°C and 100% humidity and plunged into liquid ethane using a Vitrobot Mark IV (Thermo Fisher). All data screenings were performed using a Glacios 200 kV microscope, equipped with a Falcon 4i direct electron detector (Thermo Fisher), at a magnification of 190,000×, corresponding to a calibrated pixel size of 0.732 Å.

### 4.3 Data collection

The best grids were selected for data collection on a Titan Krios 300 kV microscope (Thermo Fisher), equipped with a Gatan's BioQuantum energy filter and K3 direct electron detector. Movies were collected with EPU (Thermo Fisher) at a magnification of 130,000×, corresponding to a calibrated pixel size of 0.647 Å, with a total dose of 53 e^−^ Å^−2^ distributed across 50 frames. The defocus range was set between −0.8 and −2.4 μm in 0.2 μm intervals. A total of 37,875 movies recorded in TIFF format were collected and processed with CryoSPARC, V4.6 (Punjani et al. 2017).

### 4.4 Data processing

The workflow for data processing is illustrated in Figure S2. Movies were gain‐normalized, aligned, and dose‐weighted using patch motion correction, followed by patch CTF estimation. After exposure curation, 34,623 micrographs were accepted. An initial set of particles was picked from 5,000 micrographs using blob picking, followed by multiple rounds of 2D classification to generate templates for template picking. Forty‐five 2D classes (258,818 particles) were then selected as templates to train a Topaz picking model (Bepler et al. 2019). This model was used to pick ~4.7 million particles from the entire dataset. The particles were then extracted with a box size of 256 × 256 pixels and subjected to multiple rounds of 2D classification (using 80 online‐EM iterations and a class batch size of 200) to remove junk class averages, leaving only classes with robust secondary structure features (2,955,425 particles). An initial round of ab initio model reconstruction, followed by heterogeneous refinement, resulted in 1,595,325 particles, which yielded a map with an average resolution of 4.1 Å. Subsequent rounds of 2D classifications and further heterogeneous refinement produced a set of 671,545 particles. The particles then underwent homogeneous and non‐uniform refinements, leading to a final map with an average resolution of 3.31 Å at the Fourier shell correlation (FSC) cut‐off of 0.143. The average resolution at the 0.5 FSC cut‐off was 3.76 Å.

### 4.5 Structural model building

To generate the initial structural model, the rat CPR crystal structure (PDB code 3es9, molecule A) was docked into the cryo‐EM map. The position of the FMN‐binding domain was corrected by fitting it as a separate molecule in the electron density map in Chimera (Pettersen et al. 2004). The resulting initial model was manually adjusted in Coot (Emsley et al. 2010) followed by iterative real‐space refinement and validation in Phenix (Afonine et al. 2012). The final model validation was performed using the wwPDB Validation server (https://validate-rcsb-2.wwpdb.org). The electron density map and the associated coordinate file have been deposited in the Electron Microscopy Data Bank (EMDB) and the Protein Data Bank (PDB), respectively, under the accession codes EMD‐40857 and 9EF0. A detailed description of the data collection parameters, model refinement, and validation statistics is provided in Table 1.

### 4.6 Conformational variability detection

Next, we attempted to distinguish discrete heterogeneous states of the CPR sample. The set of 2.9 million particles, previously selected through 2D classification, was subjected to ab‐initio reconstruction (1 class), homogeneous refinement (yielding a 4.9 Å resolution map), and 3D classification (without alignment, with a filter resolution of 6 Å). The maps were examined in Chimera, and the particles were regrouped into five super‐classes. Each super‐class was then subjected to further rounds of 2D classifications and 2D class selection to remove junk particles, followed by heterogeneous, homogeneous, and non‐uniform refinements. The model‐building procedure was carried out as described earlier, except that the EM structure 9EF0 was now used as the template. The workflow is illustrated in Figure S2. The resulting subsets included 671,545, 456,689, 245,055, 139,825, and 82,212 particles, with the final resolution, based on FSC at 0.143, being 3.3, 3.5, 4.3, 3.6, and 4.4 Å, respectively. The associated coordinate files showed the N5‐to‐N5 distances of 15, 16, 17, 18, and 22 Å (Figure S5).

The rounds of 2D classifications revealed a few class averages that appeared to lack density for the FMN‐binding domain. These particles were selected as new templates for particle picking, which was followed by pick inspection, particle extraction, and further 2D classifications. Twenty 2D classes (186,327 particles) were then used to train a Topaz picking model. This model picked 5.3 million particles from the entire dataset. Subsequent 2D classifications and selections retained 1.3 million particles, which were divided into three groups. Ab‐initio reconstruction and heterogeneous refinement of the first group produced 225,003 particles, which were then subjected to non‐uniform refinement, resulting in an FMN‐domain‐absent CPR map with an average resolution of 4.53 Å (Figure S6a). Processing groups 2 and 3 yielded 44,400 and 69,108 particles, respectively, with some density observed for the FMN‐binding domain in two open conformations. The maps had average resolutions of 6.44 and 6.42 Å (Figure S6b,c). To generate the initial structural models, we followed the procedure described above for 9EF0. The resulting models were adjusted in Coot and refined in Phenix.

### 4.7 Data visualization and comparison

3D volume visualization and molecular image preparation were performed using Chimera 1.19. Structural comparisons were carried out and RMSDs calculated in LSQkab, the CCP4 program suite (Winn et al. 2011), using the secondary structure matching algorithm (Krissinel and Henrick 2004).

## AUTHOR CONTRIBUTIONS

**Galina I. Lepesheva:** Conceptualization; investigation; funding acquisition; writing – original draft; methodology; validation; visualization; writing – review and editing; data curation; formal analysis. **Tatiana Y. Hargrove:** Methodology; investigation. **Yi Ren:** Data curation; resources; formal analysis; investigation; funding acquisition; writing – review and editing.

## CONFLICT OF INTEREST STATEMENT

The authors declare no conflicts of interest.

## Supporting information


**Movie S1.** Molecular breathing of CPR in the closed conformations, visualized in ChimeraX.

**Figure S1.** Crystal structure of rat CPR (1AMO, tan) superimposed with the structures of (a) FMN‐containing flavodoxins from *Desulfovibrio vulgaris* (1C7F, cyan, RMSD = 1.14 Å), *Anacystis nidulans* (1OFV, blue, RMSD = 1.08 Å), and *Clostridium beijerinckii* (5NLL, gray, RMSD = 1.17 Å). (b) FAD‐containing flavin reductase from *Escherichia coli* (1QFJ, orange, RMSC = 1.13 Å), ferredoxin/flavodoxin reductase from *Rhodobacter capsulatus* (8VNK, light gray, RMSD = 1.07 Å), and ferredoxin reductase from *Azotobacter vinelandii* (1A8P, pink, RMSD = 0.97 Å).
**Figure S2.** Workflow for cryo‐EM data processing. Representative cryo‐EM image, 2D class averages, and the reconstructed electron density maps are shown. Data were processed in CryoSPARC. A set of 671,545 particles yielded a reconstruction of a CPR map with the average resolution of 3.3 Å (FSC cut‐off of 0.143). Four other sets of particles were processed to resolutions of 3.5, 4.3, 3.6, and 4.4 Å, all revealing variations in the closed CPR conformations.
**Figure S3.** Different positions of the FMN‐binding domain in crystal structure 3ES9 and EM structure 9EF0. Rainbow color from blue to red. The N‐terminus, the αF helix (residues 212–231), and the β6 strand are marked.
**Figure S4.** 3D domain arrangement in CPR in EM structure 9EF0. Orientation and coloring are the same as in crystal structure 1JA0 in Figure 1c.
**Figure S5.** Detected by cryo‐EM variability of closed CPR conformations. In each case, the FMN‐binding domain and the rest of the 9EF0 molecule were separately fitted into the corresponding electron density map in Chimera, the resulting structures were inspected in Coot and refined in Phenix. The N5‐to‐N5 distances are marked. The electron density maps are presented as a gray mesh. The insets show the corresponding FSC plots for the final map reconstruction with the indicated average resolution at the FSC cut‐off of 0.143.
**Figure S6.** Detected by cryo‐EM variability of open CPR conformations. (a) Molecules with no density for the FMN‐binding domain (225,003 particles). (b) Molecules with density corresponding to the FMN‐binding domain in the open 3FJO‐like CPR conformation (44,400 particles). (c) Molecules with density corresponding to the FMN‐binding domain in the open but rotated conformation (69,108 particles). Left: 2D class averages. Right: the FSC plots for the final map reconstruction, with the indicated average resolution at the FSC cut‐off of 0.143.

## Data Availability

The data that support the findings of this study are openly available in the Electron Microscopy Data Bank and PDB at https://doi.org/10.2210/pdb9EF0/pdb, reference numbers EMD‐40857 and 9EF0, respectively.
