# Supplementary material for: Cryo‐EM reveals an ensemble of cytochrome P450 reductase conformations in solution
Source: Protein Sci. 2026 Jan 21;35(2):e70448. doi: 10.1002/pro.70448 (PMC12820792; doi:10.1002/pro.70448)
Supplement: Supplementary file 2 — Figure S1. Crystal structure of rat CPR (1AMO, tan) superimposed with the structures of (a) FMN‐containing flavodoxins from Desulfovibrio vulgaris (1C7F, cyan, RMSD = 1.14 Å), Anacystis nidulans (1OFV, blue, RMSD = 1.08 Å), and Clostridium beijerinckii (5NLL, gray, RMSD = 1.17 Å). (b) FAD‐containing flavin reductase from Escherichia coli (1QFJ, orange, RMSC = 1.13 Å), ferredoxin/flavodoxin reductase from Rhodobacter capsulatus (8VNK, light gray, RMSD = 1.07 Å), and ferredoxin reductase from Azotobacter vinelandii (1A8P, pink, RMSD = 0.97 Å). Figure S2. Workflow for cryo‐EM data processing. Representative cryo‐EM image, 2D class averages, and the reconstructed electron density maps are shown. Data were processed in CryoSPARC. A set of 671,545 particles yielded a reconstruction of a CPR map with the average resolution of 3.3 Å (FSC cut‐off of 0.143). Four other sets of particles were processed to resolutions of 3.5, 4.3, 3.6, and 4.4 Å, all revealing variations in the closed CPR conformations. Figure S3. Different positions of the FMN‐binding domain in crystal structure 3ES9 and EM structure 9EF0. Rainbow color from blue to red. The N‐terminus, the αF helix (residues 212–231), and the β6 strand are marked. Figure S4. 3D domain arrangement in CPR in EM structure 9EF0. Orientation and coloring are the same as in crystal structure 1JA0 in Figure 1c. Figure S5. Detected by cryo‐EM variability of closed CPR conformations. In each case, the FMN‐binding domain and the rest of the 9EF0 molecule were separately fitted into the corresponding electron density map in Chimera, the resulting structures were inspected in Coot and refined in Phenix. The N5‐to‐N5 distances are marked. The electron density maps are presented as a gray mesh. The insets show the corresponding FSC plots for the final map reconstruction with the indicated average resolution at the FSC cut‐off of 0.143. Figure S6. Detected by cryo‐EM variability of open CPR conformations. (a) Molecules with no density for t [file PRO-35-e70448-s001.pdf]

Supplementary Material

Cryo-EM reveals an ensemble of cytochrome P450 reductase conformations in solution

Galina I. Lepesheva[1,2], Tatiana Y. Hargrove[1], Yi Ren[1,2]

[1]Department of Biochemistry, Vanderbilt University School of Medicine, Nashville, TN, USA.
[2]Center for Structural Biology, Vanderbilt University, Nashville, TN, USA

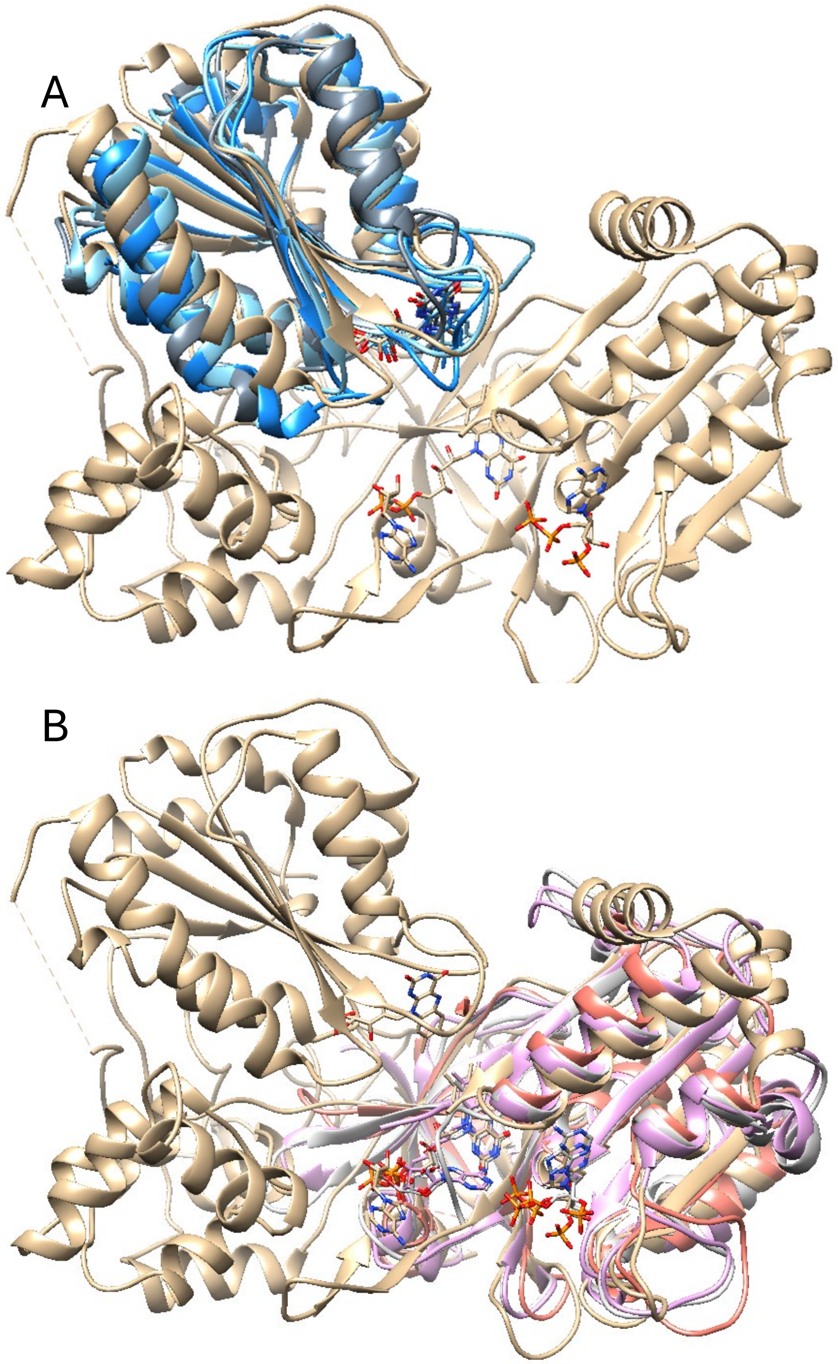

Figure S1. Crystal structure of rat CPR (1AMO, tan) superimposed with the structures of (***A***) FMN-containing flavodoxins from *Desulfovibrio vulgaris* (1C7F, cyan, RMSD =1.14Å), *Anacystis nidulans* (1OFV, blue, RMSD=1.08Å), and *Clostridium beijerinckii* (5NLL, gray, RMSD=1.17Å); (***B***) FAD-containing flavin reductase from *Escherichia coli* (1QFJ, orange, RMSC=1.13Å), ferredoxin/flavodoxin reductase from *Rhodobacter capsulatus* (8VNK, light gray, RMSD=1.07Å), and ferredoxin reductase from *Azotobacter vinelandii* (1A8P, pink, RMSD=0.97Å).

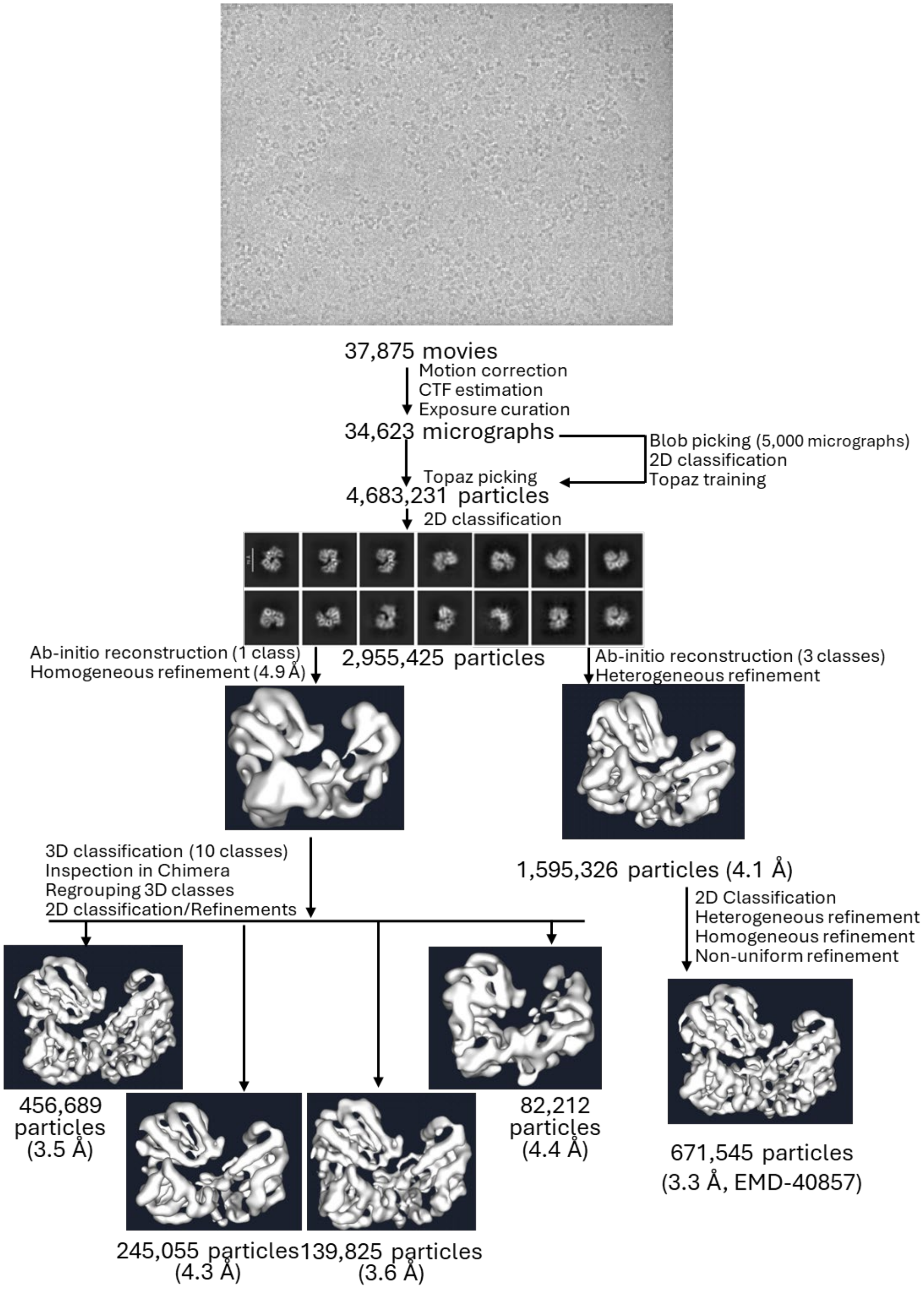

Figure S2. **Workflow for cryo-EM data processing**. Representative cryo-EM image, 2D class averages, and the reconstructed electron density maps are shown. Data were processed in CryoSPARC. A set of 671,545 particles yielded a reconstruction of a CPR map with the average resolution of 3.3 Å (FSC cut-off of 0.143). Four other sets of particles were processed to resolutions of 3.5, 4.3, 3.6, and 4.4 Å, all revealing variations in the closed CPR conformations.

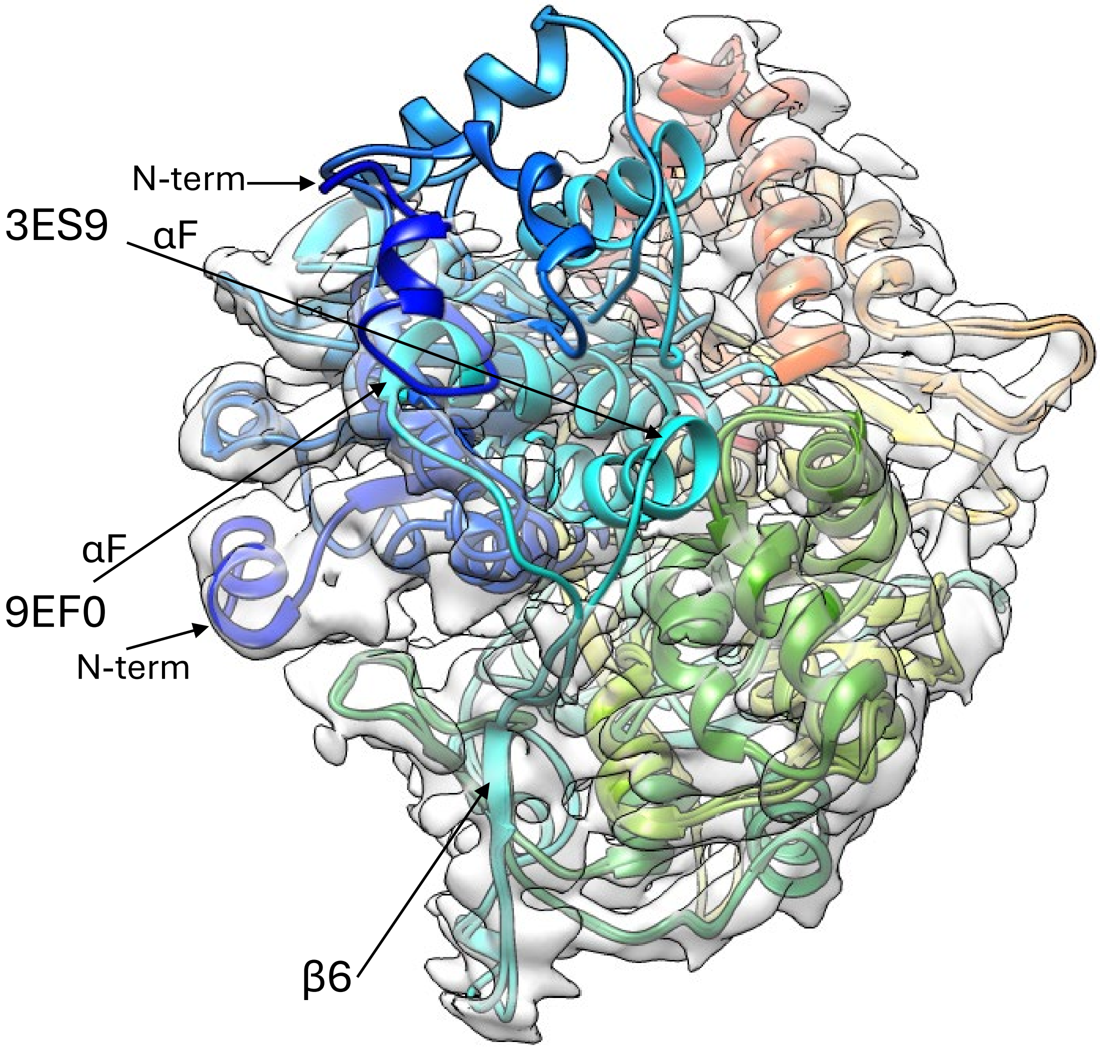

Figure S3. Different positions of the FMN-binding domain in crystal structure 3ES9 and EM structure 9EF0. Rainbow color from blue to red. The N-terminus, the αF helix (residues 212-231), and the β6 strand are marked.

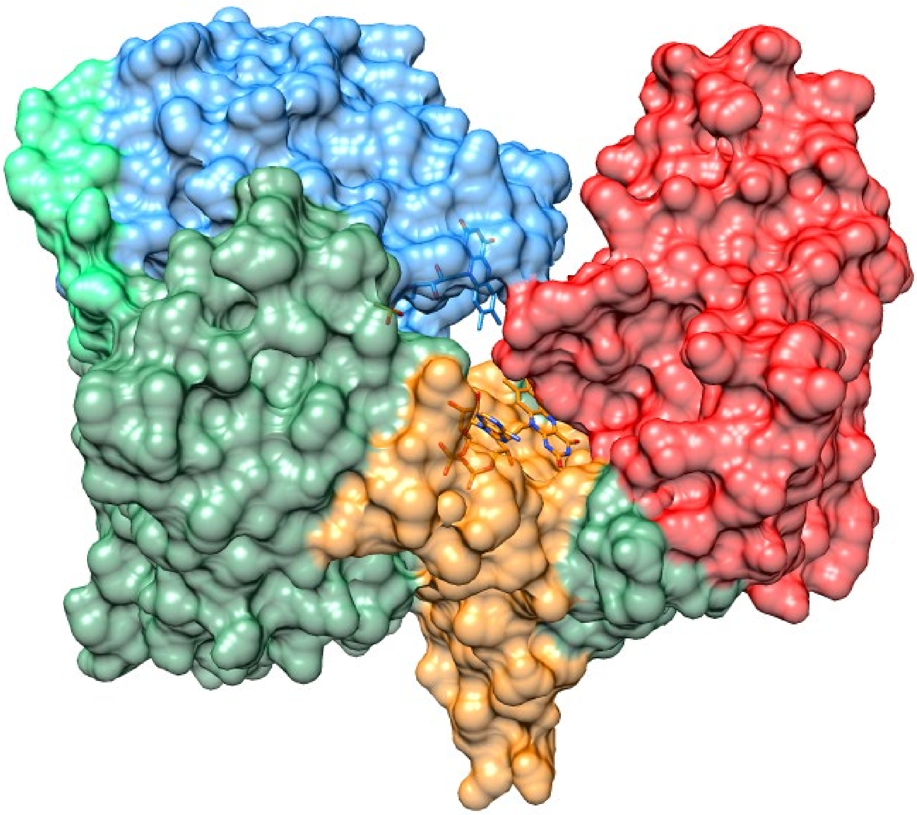

Figure S4. 3D domain arrangement in CPR in EM structure 9EF0. Orientation and coloring are the same as in crystal structure 1JA0 in Figure 1C.

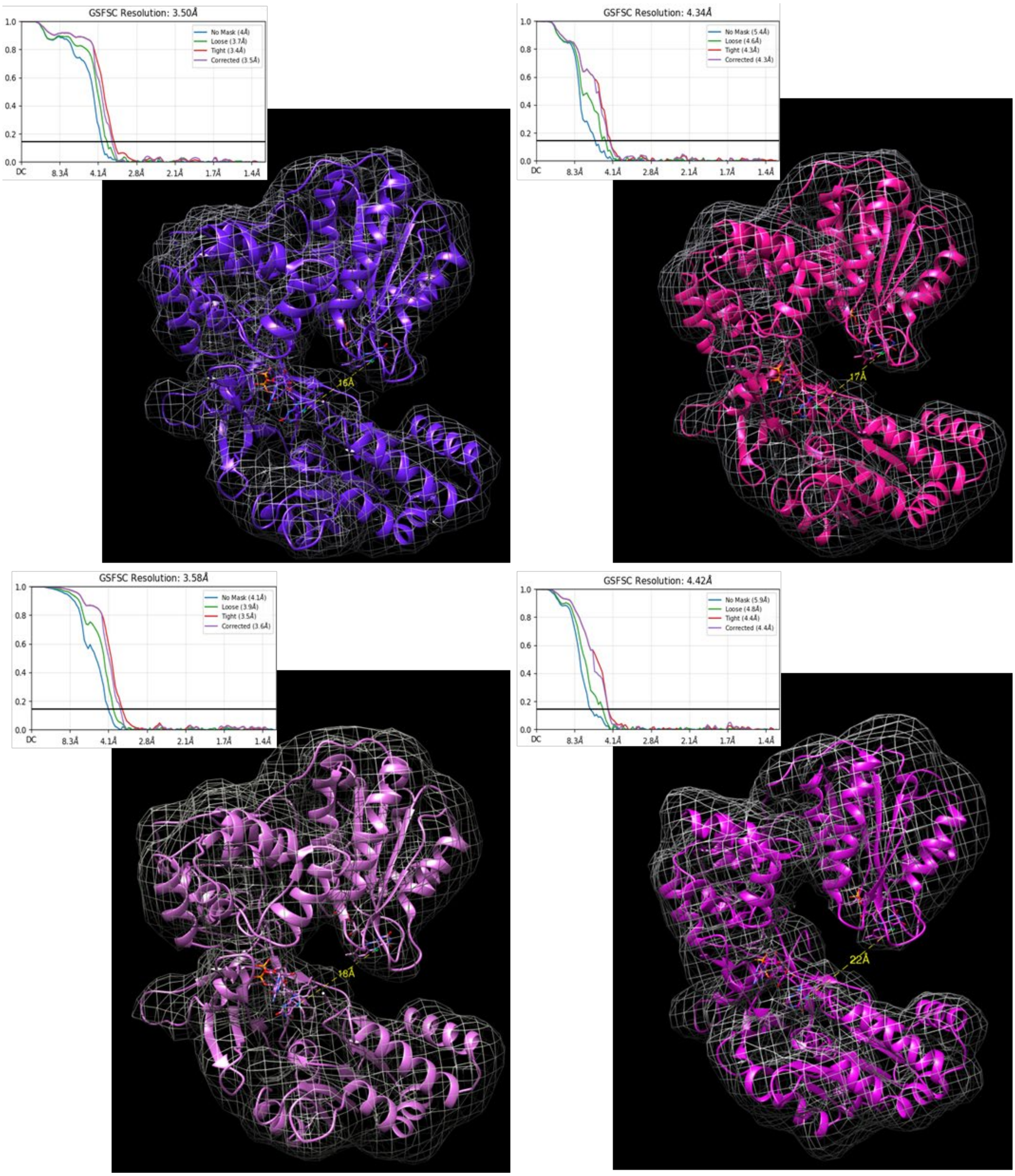

Figure S5. **Detected by cryo-EM variability of closed CPR conformations**. In each case, the FMN-binding domain and the rest of the 9EF0 molecule were separately fitted into the corresponding electron density map in Chimera, the resulting structures were inspected in Coot and refined in Phenix. The N5-to-N5 distances are marked. The electron density maps are presented as a gray mesh. The insets show the corresponding FSC plots for the final map reconstruction with the indicated average resolution at the FSC cut-off of 0.143.

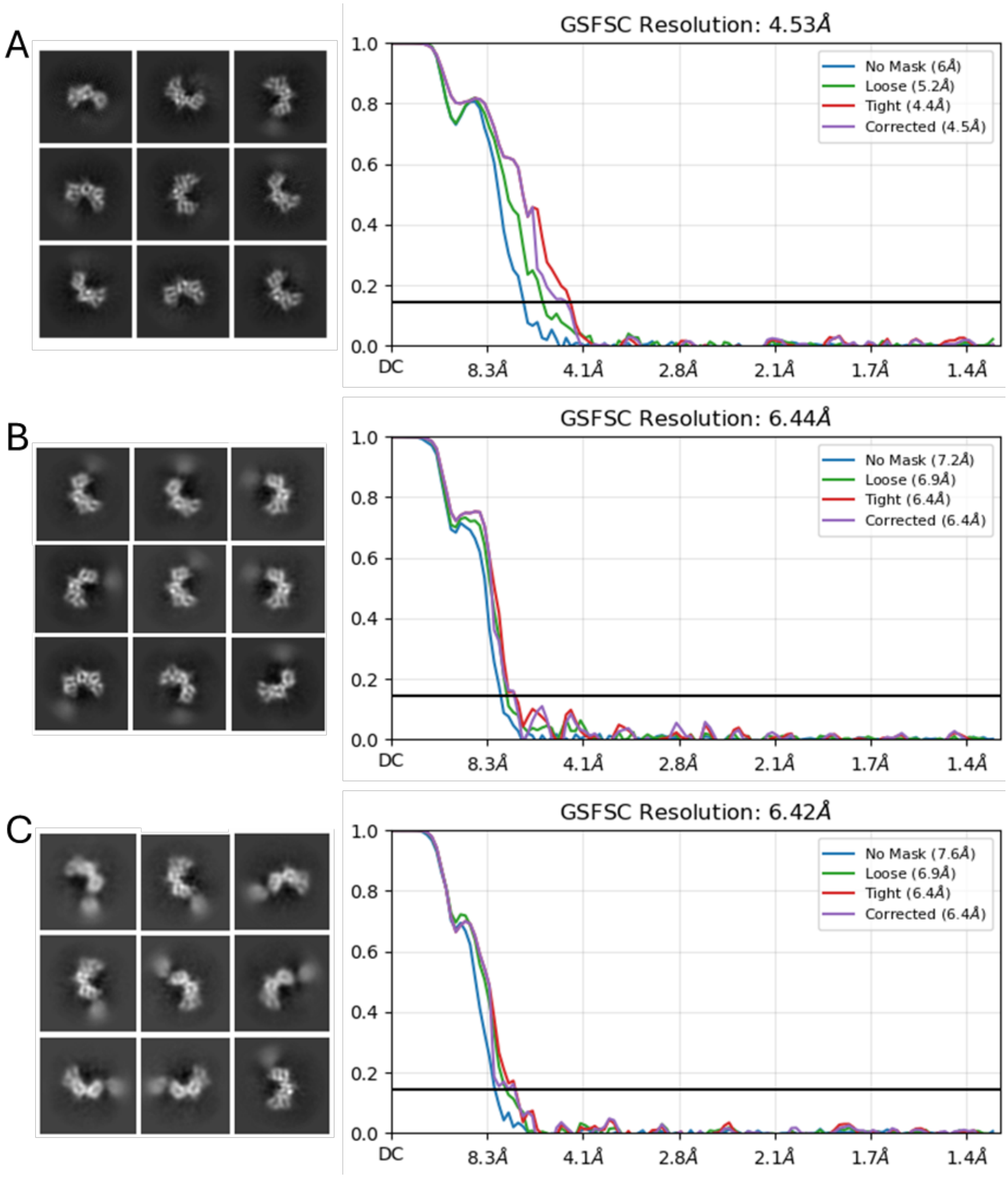

Figure S6. **Detected by cryo-EM variability of open CPR conformations**. ***A***. Molecules with no density for the FMN-binding domain (225,003 particles). ***B***. Molecules with density corresponding to the FMN-binding domain in the open 3FJO-like CPR conformation (44,400 particles). ***C***. Molecules with density corresponding to the FMN-binding domain in the open but rotated conformation (69, 108 particles). Left: 2D class averages. Right: the FSC plots for the final map reconstruction, with the indicated average resolution at the FSC cut-off of 0.143.
